# Intestinal metabolites and the risk of autistic spectrum disorder: A two-sample Mendelian randomization study

**DOI:** 10.3389/fpsyt.2022.1034214

**Published:** 2023-01-12

**Authors:** Deyang Liu, Dengyin Bu, Hong Li, Qingsong Wang, Xudong Ding, Xiaolu Fang

**Affiliations:** ^1^Department of Rehabilitation Medicine, Xiangyang No.1 People’s Hospital, Hubei University of Medicine, Xiangyang, China; ^2^Department of Psychiatric Medicine, Xiangyang Central Hospital, Hubei University of Arts and Science, Xiangyang, China; ^3^Department of Clinical Laboratory, Xiangyang No.1 People’s Hospital, Hubei University of Medicine, Xiangyang, China

**Keywords:** autistic spectrum disorder, intestinal metabolites, Mendelian randomization, serotonin, gut-brain axis

## Abstract

**Background:**

Observational studies have reported a strong association between autistic spectrum disorder (ASD) and intestinal metabolites. However, it is unclear whether this correlation is causally or violated by confounding or backward causality. Therefore, this study explored the potential causal relationship between intestinal metabolites and dependent metabolites on ASD.

**Methods:**

We used a two-sample Mendelian random analysis and selected variants closely related to intestinal flora-dependent metabolites as instrumental variables. MR-Egger, inverse variance weighted (IVW), MR-PRESSO, maximum likelihood, and weighted median were performed to reveal their causal relationships. Ten metabolites were studied, which included trimethylamine-N-oxide, betaine, carnitine, choline, glutamate, kynurenine, phenylalanine, serotonin, tryptophan, and tyrosine. Sensitivity tests were also performed to evaluate the robustness of the MR study.

**Results:**

The IVW method revealed that serotonin may increase the ASD risk (OR 1.060, 95% CI: 1.006–1.118), while choline could decrease the ASD risk (OR 0.925, 95% CI: 0.868–0.988). However, no definite causality was observed between other intestinal metabolites (e.g., trimethylamine-N-oxide, betaine, and carnitine) with ASD. Additionally, neither the funnel plot nor the MR-Egger test showed horizontal pleiotropy, and the MR-PRESSO test found no outliers. Cochran’s Q test showed no significant heterogeneity among the studies, suggesting the robustness of the study.

**Conclusion:**

Our study found potential causality from intestinal metabolites on ASD. Clinicians are encouraged to offer preventive measures to such populations.

## 1. Introduction

In the groundbreaking clinical description based on 11 boys with emotional contact disorder, Kanner first identified autistic spectrum disorder (ASD) as a coherence disorder in 1943 (1). With the developing understanding of cognitive and behavioral disorders as organic and cerebral pathologies, the concept of this disease in psychiatry has changed significantly. In the 1960s and 1970s, ASD was considered a form of psychosis similar to childhood schizophrenia. Then, it was primarily related to the parenting style (2). Epidemiological studies have shown that the prevalence of ASD was about 4 out of 10,000 children. However, with the passage of time and the update of diagnostic criteria, this estimate had increased from 1/1,000 in 1988 to 1/150–1/200 in 2002 (3). The prevalence was 16.8 per 1,000 children aged 8 years (4). Additionally, with the continuous development of technology, understanding of the mechanism of ASD had broadened. For example, some rare genetic or biomedical diseases, such as phenylketonuria, may lead to ASD (5). Although those various potential interventions have been supported by quality research, only a few pharmacological interventions have been approved by FDA for ASD. This mainly arose due to the absence of adequate randomized controlled studies. Moreover, most of these drugs target maladaptive behavior instead of core symptoms. Finding modifiable risk factors for ASD is of great significance.

The Connectivome Theory proposed by Leonardo et al. shows that ASD is also related to many body devices such as the immune system, motor system and brain-gut axis (6). As a unique ecosystem, the intestinal flora is rich in numerous species and widely participate in various physiological activities essential to human health. Accumulating evidence from observational studies or animal studies suggested that intestinal flora was correlated with various human diseases, including various cancers, cardiovascular diseases, autoimmune diseases, and neuropsychiatric diseases. Intestinal flora could affect brain activity such as behavior by regulating the intestinal metabolites. The research of intestinal flora regulating brain and behavior involves many aspects, such as the intestinal nervous system (7), neuroimaging (8), the interaction between intestinal flora and host (9, 10), and intestinal flora-intestinal brain axis (11–13). These studies demonstrated that intestinal flora affects the brain and associated behavior through the intestinal-brain axis. Additionally, it has been reported that intestinal flora is closely related to ASD (14). However, previous associations were suggested by observational studies. Since intestinal flora could be altered with diet behavior change or diseases, it remained unclear whether the association was causal or not. Besides, whether the association was violated by reverse causality or confounding remained undetermined.

As an emerging technique, Mendelian randomization uses the genetic variation of risk factors to evaluate the causality between risk factors and specific diseases (13, 15, 16). There were large amounts of MR analyses successfully revealing the potential risk factors for many neuropsychiatric diseases (17, 18). Mendelian randomization is conceptually similar to a prospective randomized controlled trial (RCT), while the Mendelian randomization method can be conducted retrospectively. In addition, since all genetic variations are determined during pregnancy and occur before the onset of the disease, Mendelian randomization can avoid non-differential measurement error and potential deviation. With large sample sizes in GWAS data booming, MR is a suitable statistical analysis to reveal the causal effect of human intestinal metabolites on ASD.

Herein, this study discusses the potential causality between internal metabolites and ASD through Mendelian randomization analysis. Our findings can fill the gaps between human intestinal metabolites and ASD and shed light on ASD prevention through diet behavior management or drug administration.

## 2. Materials and methods

### 2.1. Ethical approval, data availability, and report

All data used in this study were acquired from public databases, which had been approved by their respective committees. Thus, this study requires no further ethical approval.

### 2.2. Data source

A total of 10 intestinal metabolites were noted in our study, including trimethylamine-N-oxide, betaine, carnitine, choline, glutamate, kynurenine, phenylalanine, serotonin, tryptophan, and tyrosine. These intestinal metabolites play important roles in maintaining healthy neuropsychiatric function (19, 20), thus their causal effects on ASD are most concerning. Data about the 10 intestinal metabolites were acquired from the results of a genome-wide association study (GWAS) based on the pooled data from the human metabolome conducted among 2,076 European participants in the Framingham Heart Study (21).

The data on ASD was obtained from the dataset published by the cross-disease group of the psychiatric genomics federation. The sample size was 61,220, including 33,332 patients with ASD and 27,888 control population. All participants in the above two GWAS were of European ancestry.

The instrument variables were identified in a GWAS of human intestinal metabolites with the following standard: (1) to obtain more SNPs as instrument variables, we selected SNPs correlated with the risk factors at a relatively relaxed statistical significance threshold (*P* < 5 × 10^–5^), (2) to remove SNPs at linkage disequilibrium *R*^2^ > 0.001 based on European ancestry reference data from the 1,000 Genomes project, and (3) to calculate the F statistic for each SNP and discard weak instrumental SNPs (*F* < 10). The instrument variables were used to investigate the causal effect of metabolome on ASD. That is, if a causal effect was found from instrument variables on ASD, the causal effect from human intestinal metabolites on ASD can be concluded.

### 2.3. Statistical analysis

Two-sample MR package of R software was used for Mendelian randomization analysis. In this study, we applied several MR approaches, including the inverse-variance weighted (IVW) method, MR-Egger, MR-PRESSO, maximum likelihood, and weighted median. The traditional IVW method was used as the main finding to estimate the association between intestinal metabolites and ASD. However, since IVW estimation may be affected by invalid instrument deviation or pleiotropy, the effectiveness and robustness of IVW results were tested using other methods, including MR-Egger, MR-PRESSO, maximum likelihood, and weighted median. To reduce the deviation caused by horizontal pleiotropy (affecting the results through causal pathways rather than exposure), MR-PRESSO (pleiotropy residuals and outliers) was used to detect extensive horizontal pleiotropy in all results. Outliers identified by MR-PRESSO would be discarded and MR analyses would be re-performed. Finally, to evaluate the robustness of the results, heterogeneity and pleiotropy tests were carried out for the statistically significant results, including the MR-Egger intercept test and modified Cochran’s Q statistics. A *p*-value of > 0.05 is expected in heterogeneity and pleiotropy analyses to support our study is unbiased. Funnel plots and leave-one-out analyses were also performed for significant MR results. Symmetrical funnel plots would be expected, which can reflect the absence of outliers. The perfect results of leave-one-out analyses would be for all estimates to remain similar and significant after each SNP is removed.

All statistical tests were conducted in a two-tailed manner. Since the results were binary variables, the estimated value of the effects was further converted into an odds ratio (OR) to demonstrate the correlation of metabolites with ASD. To account for multiple testing in our study, the Bonferroni’s-corrected significance level of *P* < 5 × 10^–3^ (0.05 divided by 10 risk factors) was used. The *p*-value between 5 × 10^–3^ and 0.05 was considered a potential association.

## 3. Results

### 3.1. Determination of instrumental variables and their weak bias

In total, 2,117 SNPs related to intestinal metabolites (77 associated with beta-hydroxybutyric acid, 264 associated with betaine, 159 associated with carnitine, 105 associated with choline, 133 associated with glutamate, 257 associated with kynurenine, 161 associated with phenylalanine, 52 associated with propionic acid, 216 associated with serotonin, 293 associated with trimethylamine-N-oxide, 170 associated with tryptophan, and 230 associated with tyrosine) were extracted from GWAS and used as IVs (*R*^2^ < 0.001, *P* < 5 × 10^–5^). The F statistics of involved SNPs were greater than 10, indicating that the instrument variables used in our study are strong. Therefore, no weak bias was detected in the results, and the conclusions of this study were reliable (Supplementary Table 1).

### 3.2. Causal effects from intestinal metabolites on ASD risk

Results of IVW supported that serotonin could increase the ASD risk (OR = 1.060, 95% CI: 1.006–1.118). Meanwhile, the results of IVW showed that choline might reduce the ASD risk (OR = 0.925, 95% CI: 0.868–0.988). Similar results were observed in other MR analyses (Table 1). Both maximum likelihood and MR-PRESSO present similar and significant estimates, while MR-Egger and weighted median present similar but insignificant estimates. No evidence of causal effects from other intestinal metabolites on ASD was found.

**TABLE 1:** MR analysis of gut metabolites and ASD.

Outcome	Exposure	MR.Egger				Inverse.variance.weighted				Maximum.likeli hood				Weighted.median				MR-PRESSO				Pleiotropy	Heterogeneity
		OR	CI	CL	*P*	OR	CI	CL	*P*	OR	CI	CL	*P*	OR	CI	CL	*P*	OR	CI	CL	*P*	*P*	*P*
ASD	trimethylamine-N-oxide	1.009	0.892	1.142	0.886	0.996	0.948	1.046	0.861	0.996	0.948	1.046	0.860	0.981	0.902	1.067	0.651	0.992	0.967	1.018	0.500	0.718	0.923
ASD	Betaine	0.893	0.762	1.047	0.173	0.993	0.934	1.055	0.815	0.993	0.934	1.055	0.809	0.954	0.872	1.044	0.306	0.995	0.936	1.057	0.854	0.162	0.348
ASD	Carnitine	1.100	0.966	1.252	0.170	1.010	0.949	1.075	0.746	1.010	0.954	1.069	0.709	1.044	0.965	1.129	0.291	1.010	0.949	1.075	0.749	0.166	0.156
ASD	Choline	0.908	0.798	1.034	0.164	0.926	0.868	0.988	0.021	0.925	0.865	0.989	0.023	0.929	0.835	1.032	0.171	0.926	0.878	0.976	0.009	0.754	0.872
ASD	Glutamate	0.807	0.510	1.277	0.377	0.944	0.856	1.041	0.244	0.944	0.854	1.042	0.244	0.944	0.827	1.077	0.466	0.944	0.878	1.015	0.140	0.508	0.893
ASD	Kynurenine	1.059	0.863	1.298	0.585	1.039	0.970	1.112	0.280	1.041	0.983	1.102	0.167	1.023	0.937	1.118	0.603	1.039	0.970	1.112	0.288	0.849	0.007
ASD	Phenylalanine	0.897	0.805	0.999	0.058	0.956	0.896	1.020	0.174	0.954	0.898	1.014	0.125	0.927	0.850	1.010	0.082	0.955	0.895	1.019	0.187	0.160	0.281
ASD	Serotonin	1.033	0.920	1.159	0.583	1.062	1.007	1.120	0.027	1.064	1.009	1.122	0.021	1.037	0.960	1.119	0.349	1.063	1.008	1.121	0.035	0.596	0.285
ASD	Tryptophan	0.839	0.713	0.987	0.045	0.982	0.917	1.052	0.612	0.981	0.914	1.053	0.600	1.035	0.934	1.146	0.509	0.982	0.926	1.042	0.551	0.047	0.838
ASD	Tyrosine	0.978	0.937	1.021	0.338	0.994	0.960	1.030	0.742	0.994	0.965	1.024	0.689	0.984	0.946	1.023	0.436	0.994	0.960	1.030	0.745	0.232	0.080

ASD, autistic spectrum disorder; OR, odds ratio; CI, confidence interval; CL, confidence level.

### 3.3. Sensitivity analysis

To confirm the stability of the above results, MR-Egger intercept analyses and MR-PRESSO tests were performed on the involved SNP variants. No potential horizontal pleiotropy was detected (*P* > 0.05), and the funnel chart did not show any deviation in this study (Supplementary Figures 11–20). The revised Cochran’s Q statistics showed no significant heterogeneity in the effect of involved SNP (*P* = 0.90). Additionally, sensitivity analysis was conducted to verify the impact of each SNP site on the overall causality. When a single SNP was systematically removed and MR analysis was repeated, no significant difference was observed (Supplementary Figures 1–10), proving that the estimated effect cannot be explained by any single genetic tool.

## 4. Discussion

This study is the first Mendelian randomization study to systematically evaluate the causality between intestinal metabolites and ASD. Our results supported the idea that serotonin may be related to increased ASD risk, while choline may reduce ASD risk. The relationship between the two intestinal metabolites and ASD was further confirmed by sensitivity analysis. In our study, we found no evidence of causal effects from other metabolites on ASD.

Central nervous system development disorders have long been associated with intestinal metabolites, creating the so-called “gut-brain axis.” ASD is one of the most frequent neurodevelopmental disorders associated with dysregulation of the microbiota-gut-brain axis. Metabolism is one of the major drivers of microbiome-host interactions. Therefore, metabolites were hypothesized to affect the risk of ASD. Previous observational studies have suggested associations between metabolites and ASD. However, it is unclear whether intestinal metabolites cause ASD or whether ASD causes changes in intestinal metabolites. Herein, we applied two-sample MR to investigate their causal effects.

The study result was in concurrence with previous studies (i.e., serotonin may increase ASD risk). A transmission/disequilibrium test study conducted on 184 cases showed that serotonin was not related to ASD risk. Another meta-analysis, including 22 observational studies, showed that serotonin might promote ASD risk (OR = 4.6, 95% CI = 3.1–5.2) (22). Overall, the exact mechanism by which serotonin causes ASD remains unclear. However, few studies have shown that the onset of ASD may be related to the function of serotonin. For instance, serotonin can regulate neuron differentiation and migration, axon growth, myelin, and synapse formation (23). Additionally, serotonin connects the intestine with the central nervous system. It also affects neurogenesis, guiding the development and survival of post-mitotic neurons [dopamine, γ-Aminobutyric acid (GABA), and calcitonin gene-related peptide-secreting neurons] (24). However, in early childhood, the blood-brain barrier is often not completed until the age of 2 years (25). Therefore, during the fetal and neonatal period, 5-HT with a high level in the blood can enter the developing brain. These high levels may inhibit 5-HT-secreting neurons through the negative feedback mechanism and lead to a decreased serotonin concentration. Azmita et al. conducted an immunocytochemical analysis, in which they found an increase of serotoninergic axons in the cerebral cortex of patients with ASD, which indirectly confirms the above hypothesis. Nevertheless, these theories cannot explain the serotonin-mediated ASD mechanism (25). Additionally, other intestinal metabolites (e.g., choline) may reduce the ASD risk, as confirmed in other studies (26); however, some reports have suggested that choline increases ASD risk (27). Trimethylamine-N-oxide, an important molecule affecting human health, was thought to be a waste product of choline metabolism without action in our organism (28). A previous study reported that TMAO was found to increase the risk of ASD (28). However, evidence in our study supports the finding that choline reduces the risk of ASD but trimethylamine-N-oxide has no effect on ASD. Therefore, there might exist another pathway by which choline acts on ASD, and the specific mechanism remains to be explored.

In MR analyses, three assumptions should be satisfied to derive robust estimates. First, the instrument variables should be strongly related to exposure. In our study, to obtain more SNPs as instrument variables, we used a relaxed *P*-value. But the F statistics had been calculated to support the presence of strong instrument variables. Second, the instrument variables should not affect the outcomes through other pathways rather than exposure. This assumption is easy to fulfill for the randomly assigned SNPs during pregnancy. Because there were no well-established risk factors for ASD, we did not perform additional analyses to reveal the association between instrument variables and potential risk factors. Third, the instrument variables should not correlate with the outcomes, and we have checked that each SNP of the instrument variables in our study was not correlated with ASD.

In our study, we used IVW estimates as the main findings and other MR approaches as complementary analyses. For the significant estimates identified by IVW, both maximum likelihood and MR-PRESSO also derive significant estimates, but insignificant results are found in MR-Egger and the weighted median. Because MR-Egger suggests that all SNPs used in the study are invalid and the weighted median suggests that half SNPs are invalid. This basic theory causes these two approaches more difficult to derive significant estimates than the other three approaches. We have searched the literature and found support from other studies to believe in the IVW estimates and draw a significant conclusion. In addition, we did not use multiple tests corrected *P*-value in our study. Our purpose is to discover any potential risk factors for ASD, and Bonferroni’s or FDR correction would reduce our opportunity to do so. With MR analyses and sensitivity analyses, we identified that choline and serotonin were correlated with ASD risk. Further studies should be performed to discover the underlying mechanisms.

Taking the results that serotonin and choline are correlated with the risk of ASD robust, we should carry out some efficient protocols to adjust these two metabolites. Antibiotic administration is one useful method that can prevent the absorption of these two metabolites from the gastric and intestinal tracts. There were some non-absorbable antibiotics (29), for example, vancomycin, neomycin, and bacitracin. These non-absorbable antibiotics offer the advantage of killing the microbiota in the gut while avoiding the disadvantage of causing reverse effects on the human body after entering systemic circulation. Another efficient method is fecal microbiota transplant, which involves the transfer of intestinal microbiota from one human being to another. The fecal microbiota transplant is becoming more and more popular to prevent or treat many diseases. The procedure of fecal microbiota transplant is mature, and some studies have shown successful transfer of microbiota (30). All these methods might be useful to alter the concentration of serotonin and choline, and thus prevent the occurrence of ASD.

There were several strengths and novelties to our study. First, this is the first MR study to investigate the causal relationship between 10 human intestinal metabolites and the risk of an ASD. The MR framework is superior to most observational studies in addressing confounding risk factors, which makes our findings robust. Notably, the results of this study represent the relationship between intestinal metabolites and the lifetime risk of ASD, as genetic variation does not change during a lifetime. Meanwhile, since alleles are randomly classified and determined during pregnancy, this study did not observe deviation due to confusion and backward causality. Second, our findings are robust because of the large sample size. The larger the sample size, the more robust the MR findings. The GWAS of ASD enrolled more than 60,000 participants, tremendously larger than most observational studies.

The study had some limitations. First, this study included only people in Europe. So, it may not represent another ethnicity, such as people in Asia and Africa. Nevertheless, it can effectively reduce deviation caused by population stratification. Second, subgroup analysis could not be performed due to the lack of detailed clinical information. Third, this study showed the potential causality between intestinal metabolites and ASD. However, more extensive research is required to confirm the definite causality between intestinal metabolites and ASD.

## 5. Conclusion

Mendelian randomization analysis revealed that serotonin could increase the ASD risk. Therefore, managing children’s diet should be an essential component to reduce ASD risk. Additionally, due to the genetic differences among different nationalities, countries, and regions, it is necessary to explore this relationship in wider and more diverse populations.

## Data availability statement

The original contributions presented in this study are included in the article/Supplementary material, further inquiries can be directed to the corresponding author.

## Author contributions

DL and DB: study design. HL: data collection and data analysis. QW and XD: data interpretation. DL and XF: drafting manuscript. All authors take responsibility for the integrity of the data analysis and approved the final version of the manuscript.
