# Supplementary material for: Intestinal metabolites and the risk of autistic spectrum disorder: A two-sample Mendelian randomization study
Source: Front Psychiatry. 2023 Jan 12;13:1034214. doi: 10.3389/fpsyt.2022.1034214 (PMC9877426; doi:10.3389/fpsyt.2022.1034214)
Supplement: Supplementary file 2 [file Data_Sheet_1.pdf]

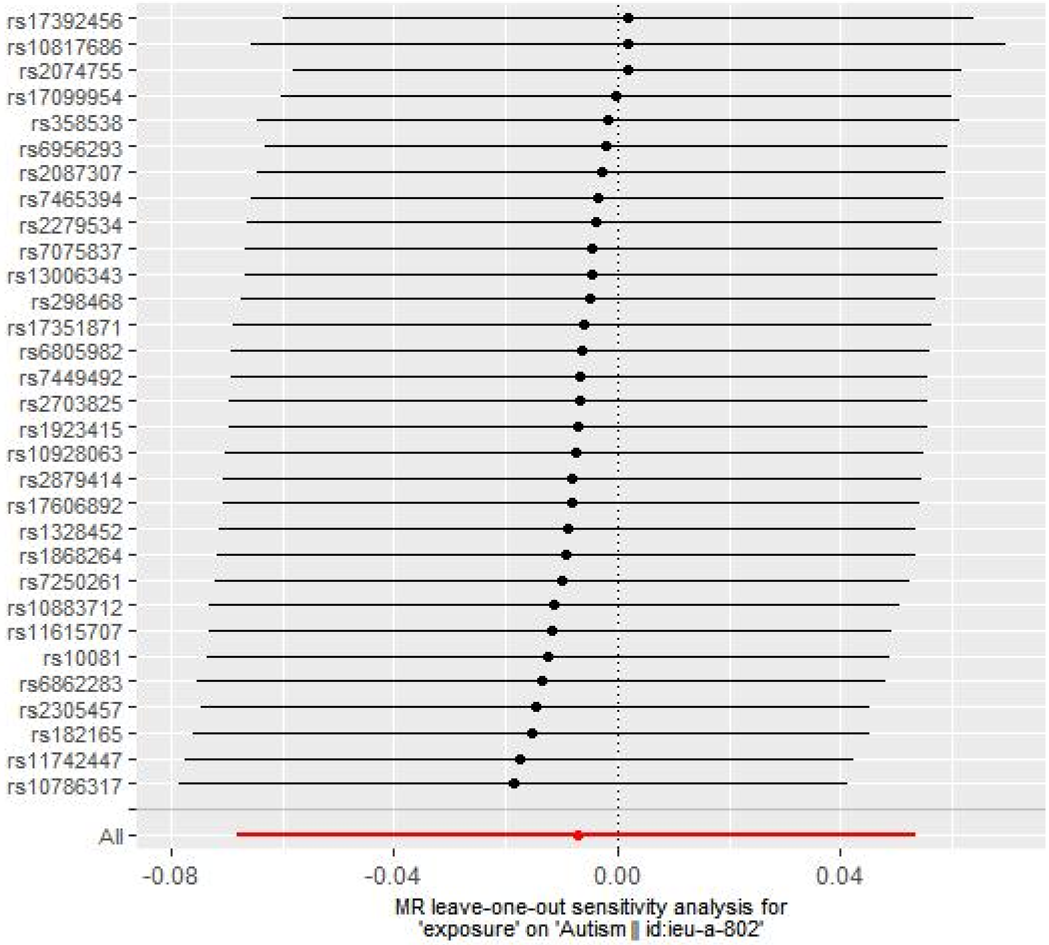

Figure 1: Leave-one-out plot to visualize causal effect of betaine on the risk of autism when leaving one SNP out.

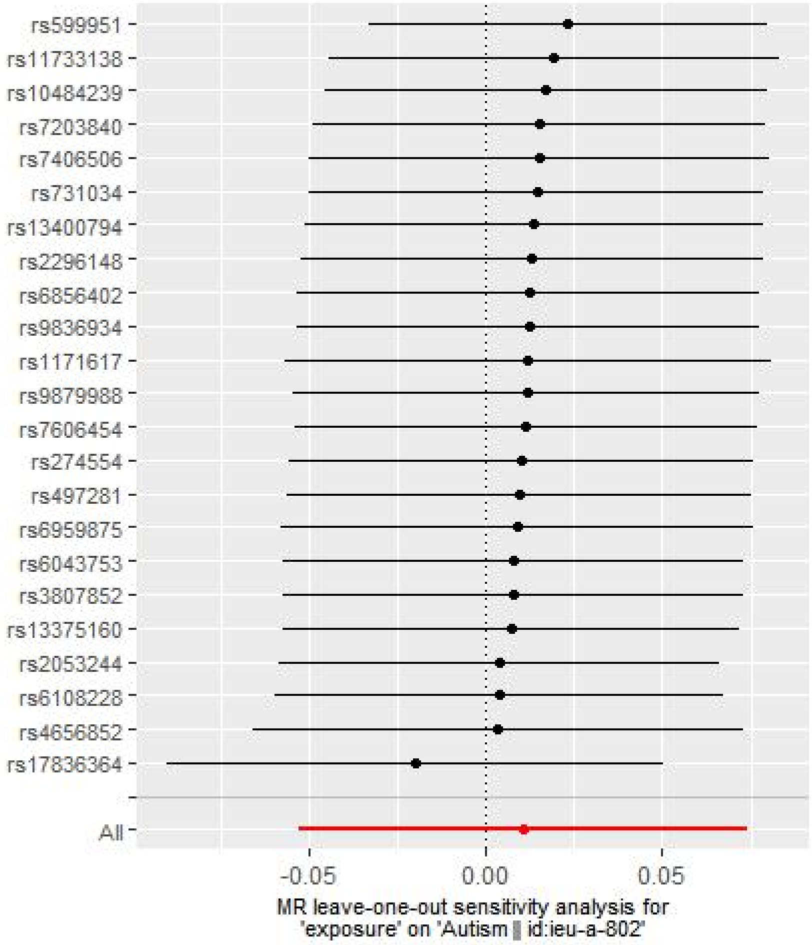

Figure 2: Leave-one-out plot to visualize causal effect of carnitine on the risk of autismwhen leaving one SNP out.

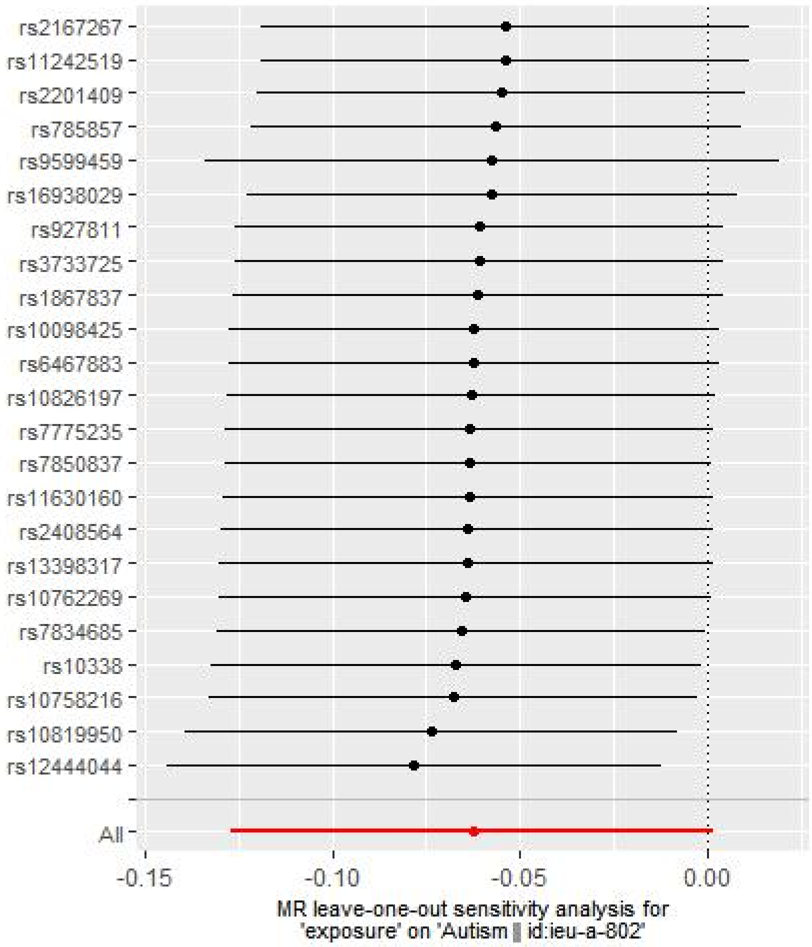

Figure 3: Leave-one-out plot to visualize causal effect of choline on the risk of autismwhen leaving one SNP out.

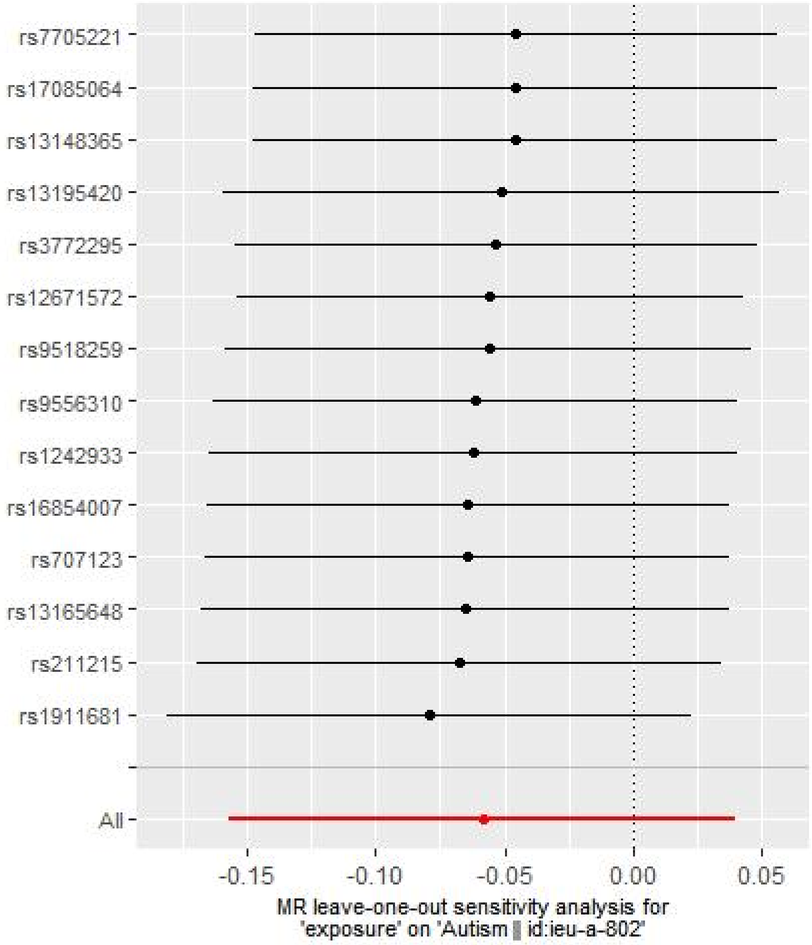

Figure 4: Leave-one-out plot to visualize causal effect of glutamate on the risk of autism when leaving one SNP out.

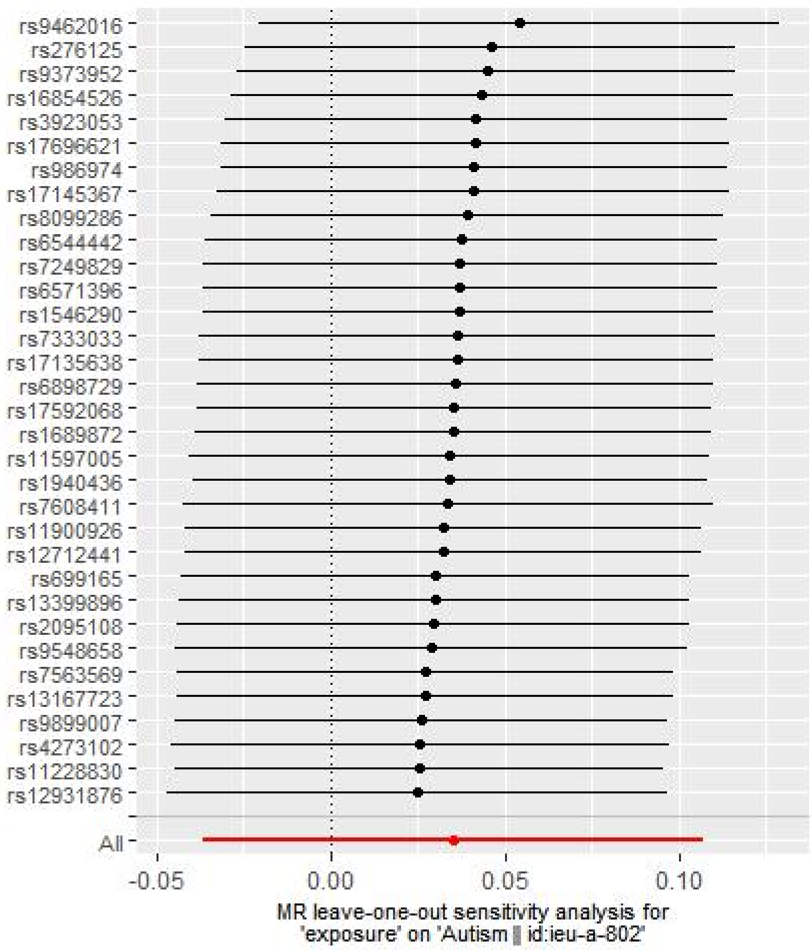

Figure 5: Leave-one-out plot to visualize causal effect of kynuremine on the risk of autism when leaving one SNP out.

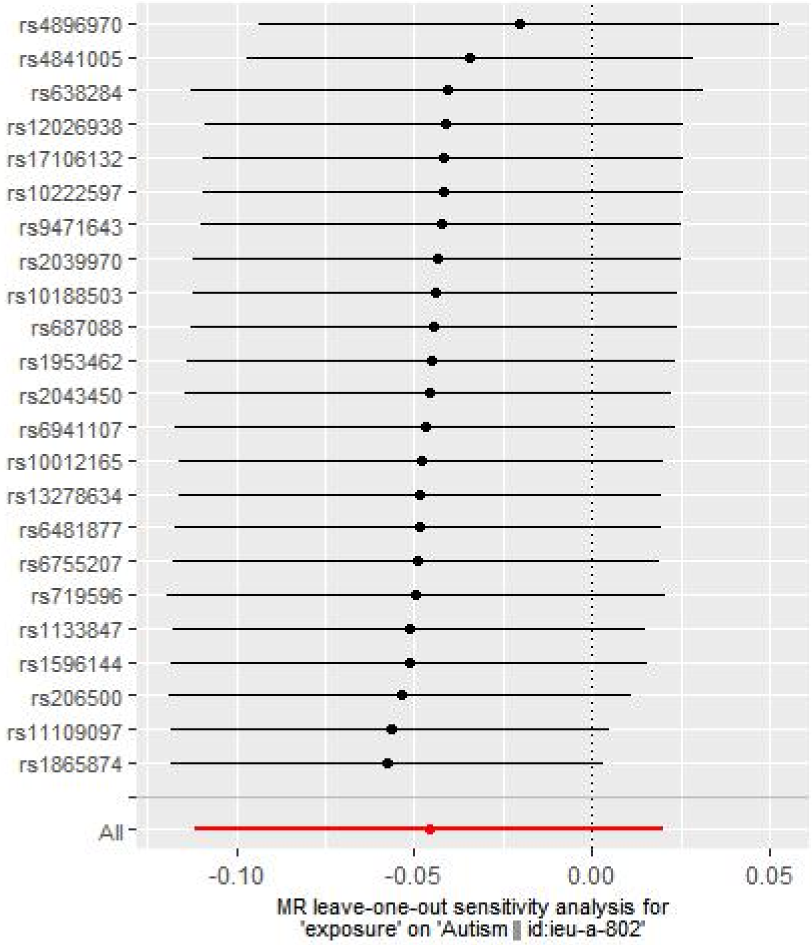

Figure 6: Leave-one-out plot to visualize causal effect of phenylalanine on the risk of autismwhen leaving one SNP out.

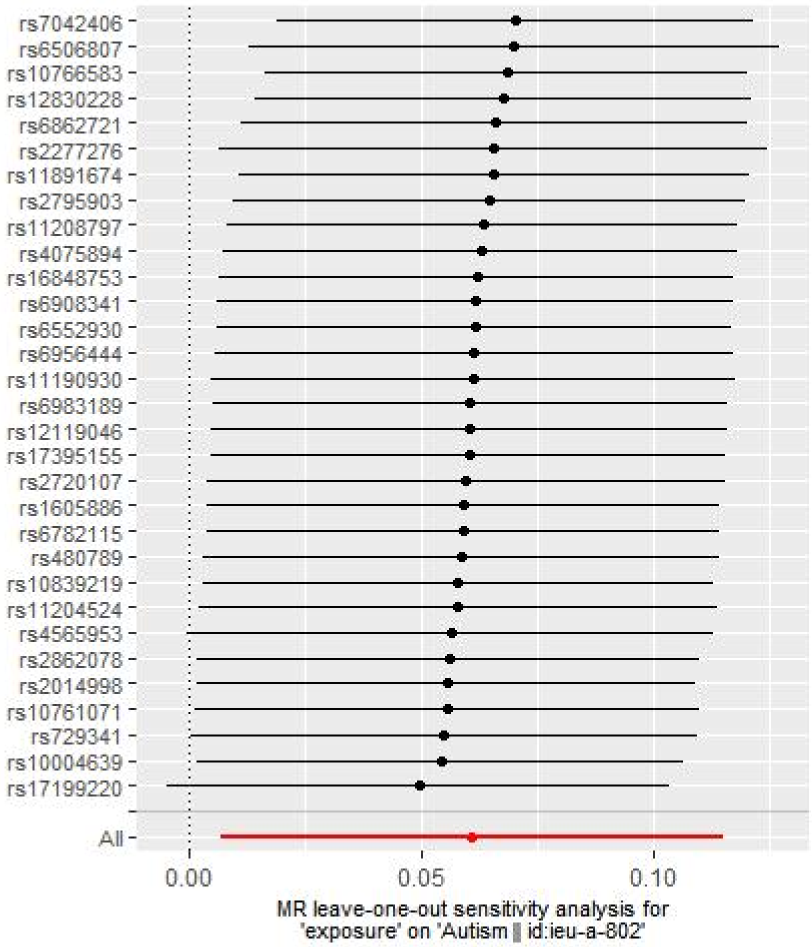

Figure 7: Leave-one-out plot to visualize causal effect of serotonin on the risk of autismwhen leaving one SNP out.

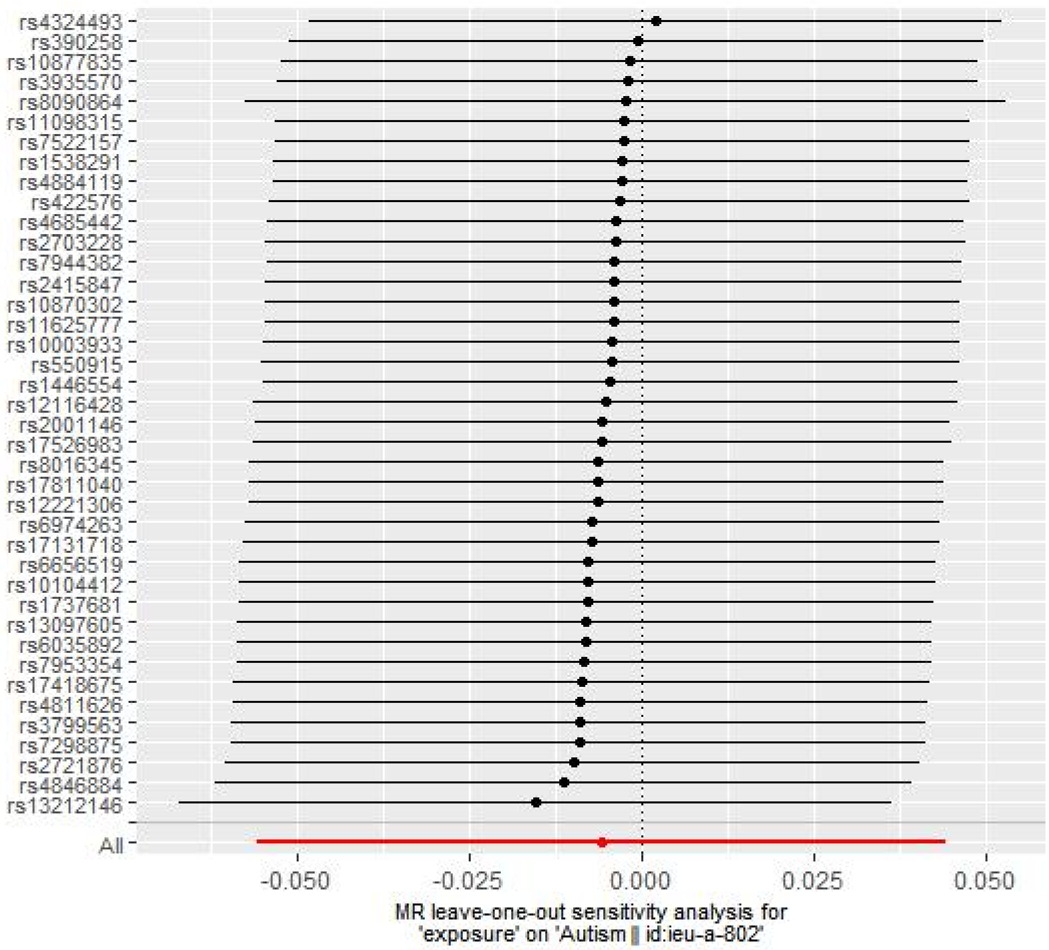

Figure 8: Leave-one-out plot to visualize causal effect of trimethylamine_N_oxide on the risk of autism when leaving one SNP out.

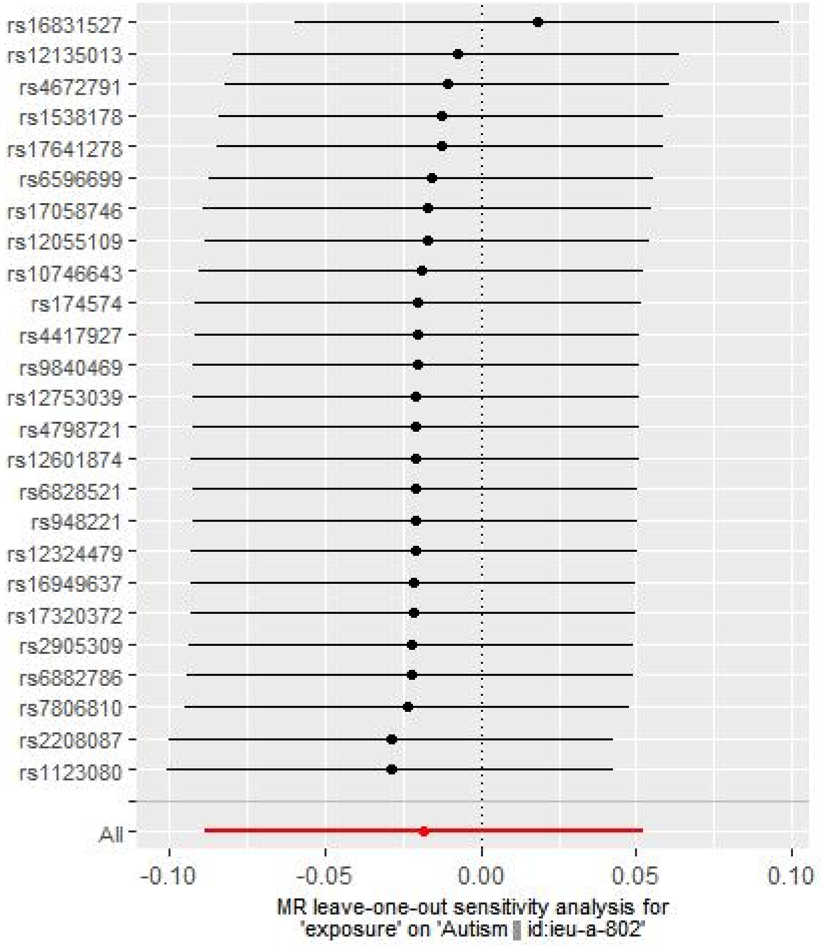

Figure 9: Leave-one-out plot to visualize causal effect of tryptophan on the risk of autism when leaving one SNP out.

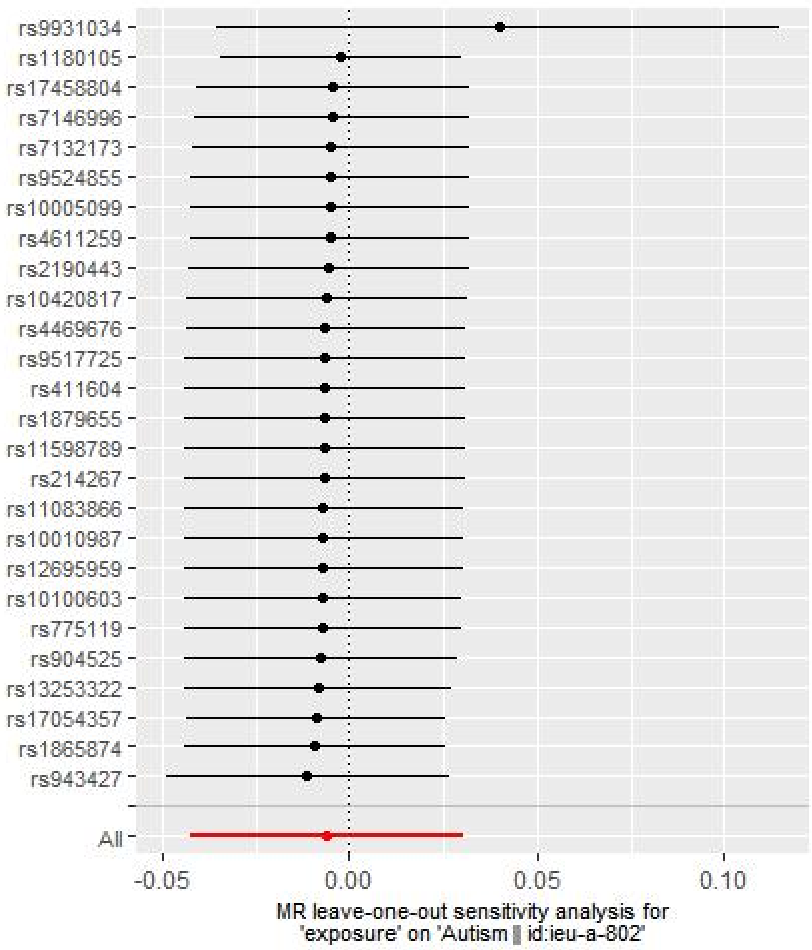

Figure 10: Leave-one-out plot to visualize causal effect of tyrosine on the risk of autism when leaving one SNP out.

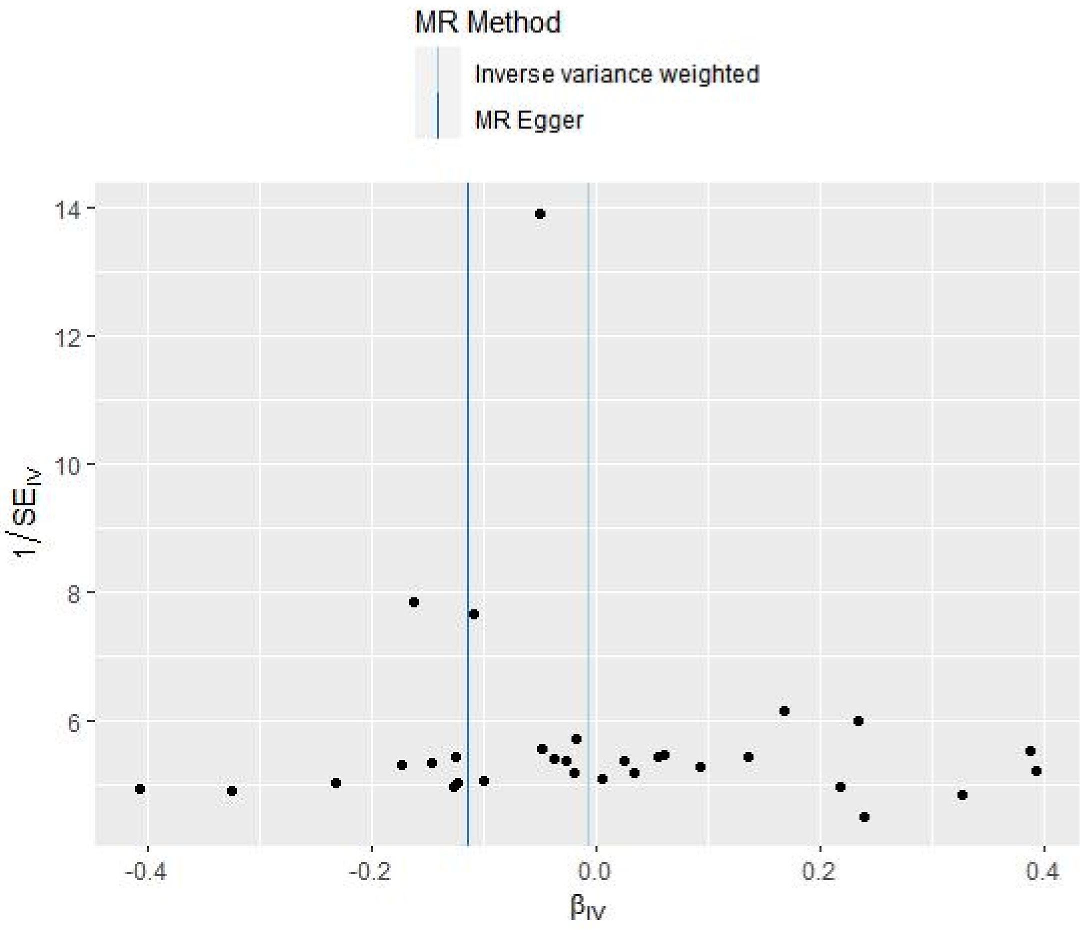

Figure 11: Funnel plots to visualize overall heterogeneity of Mendelian randomization (MR) estimates for the effect of betaine on the risk of autism.

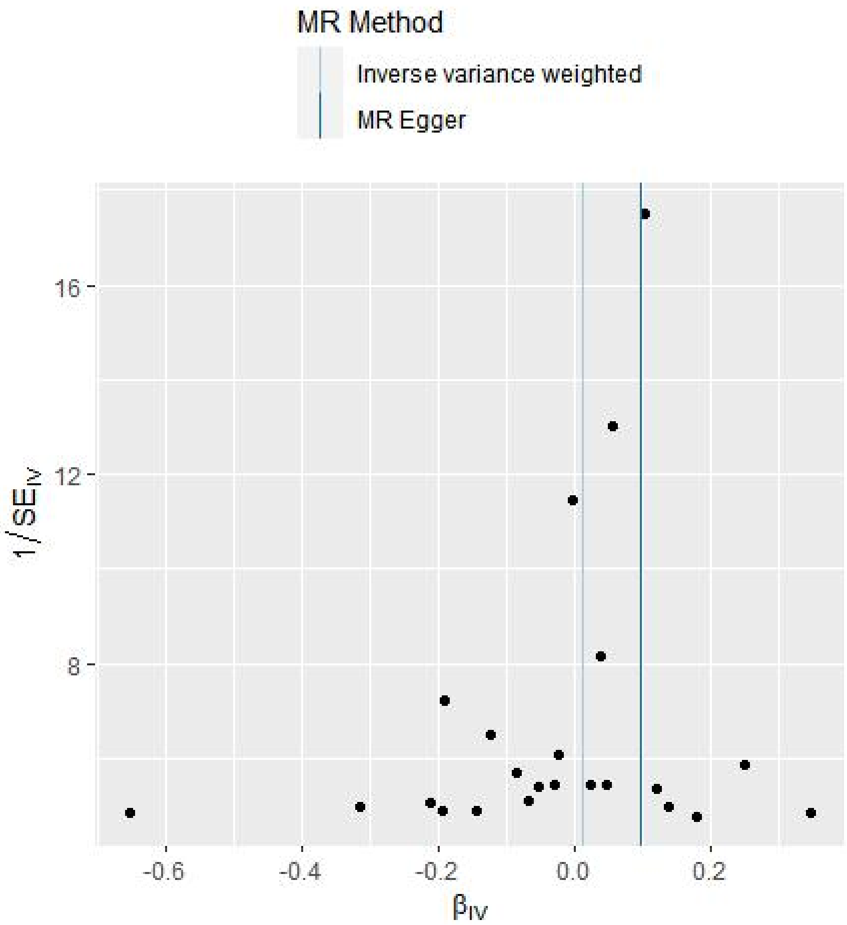

Figure 12: Funnel plots to visualize overall heterogeneity of Mendelian randomization (MR) estimates for the effect of carnitine on the risk of autism.

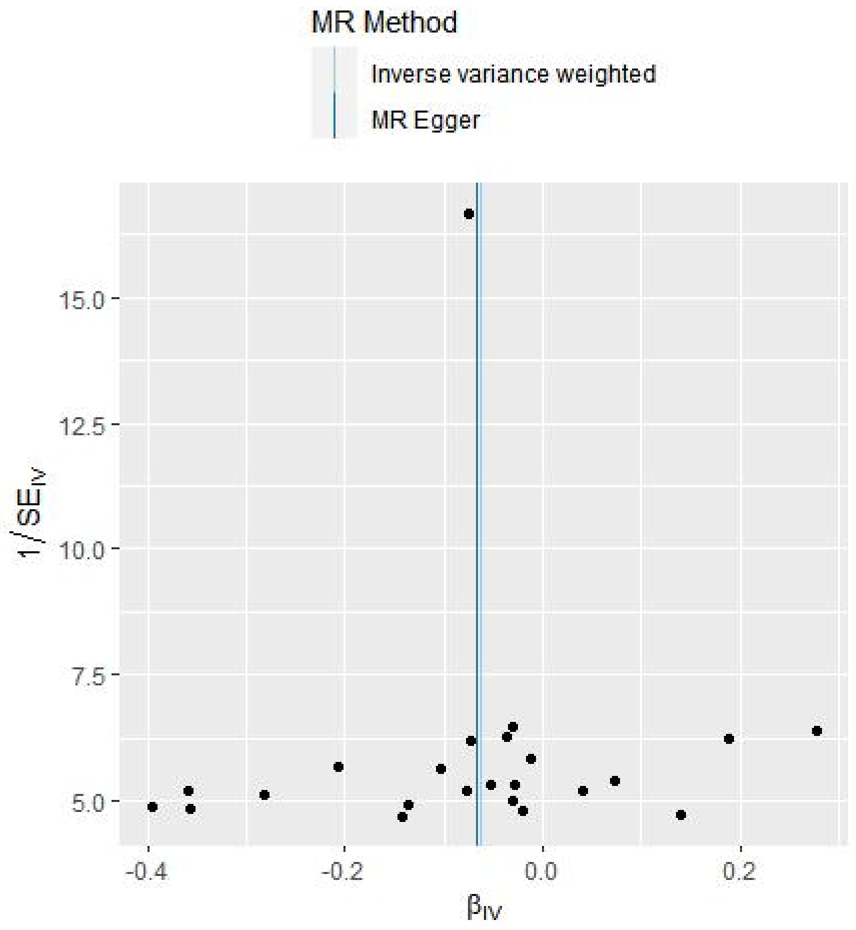

Figure 13: Funnel plots to visualize overall heterogeneity of Mendelian randomization (MR) estimates for the effect of choline on the risk of autism.

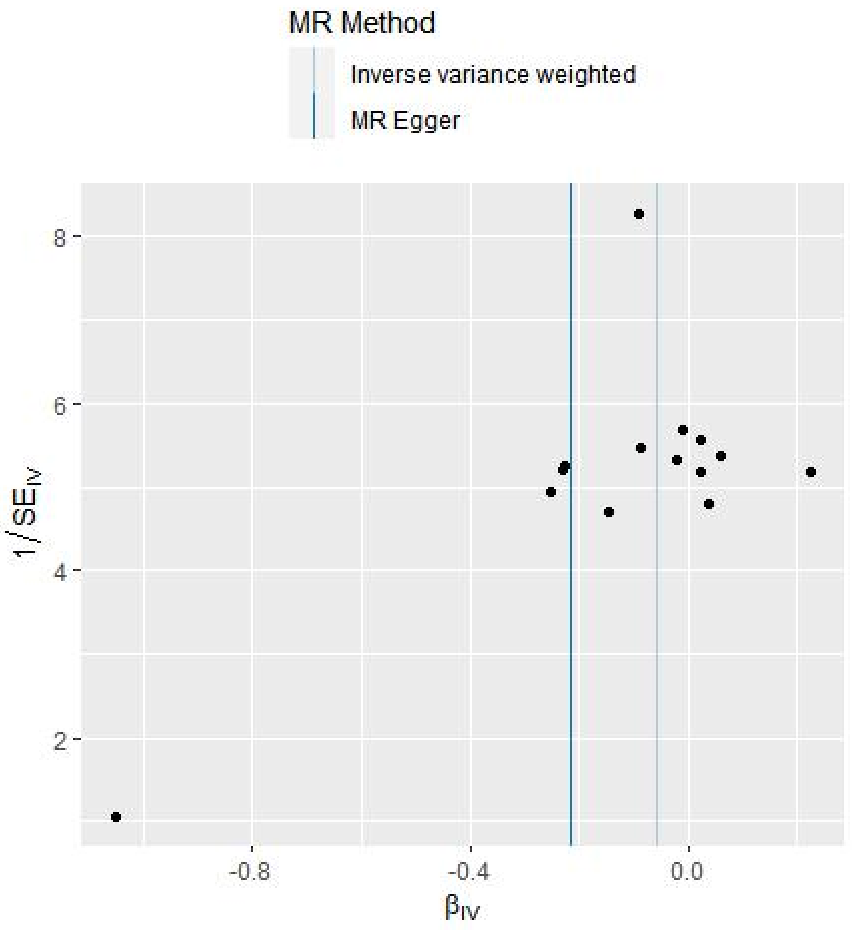

Figure 14: Funnel plots to visualize overall heterogeneity of Mendelian randomization (MR) estimates for the effect of glutamate on the risk of autism

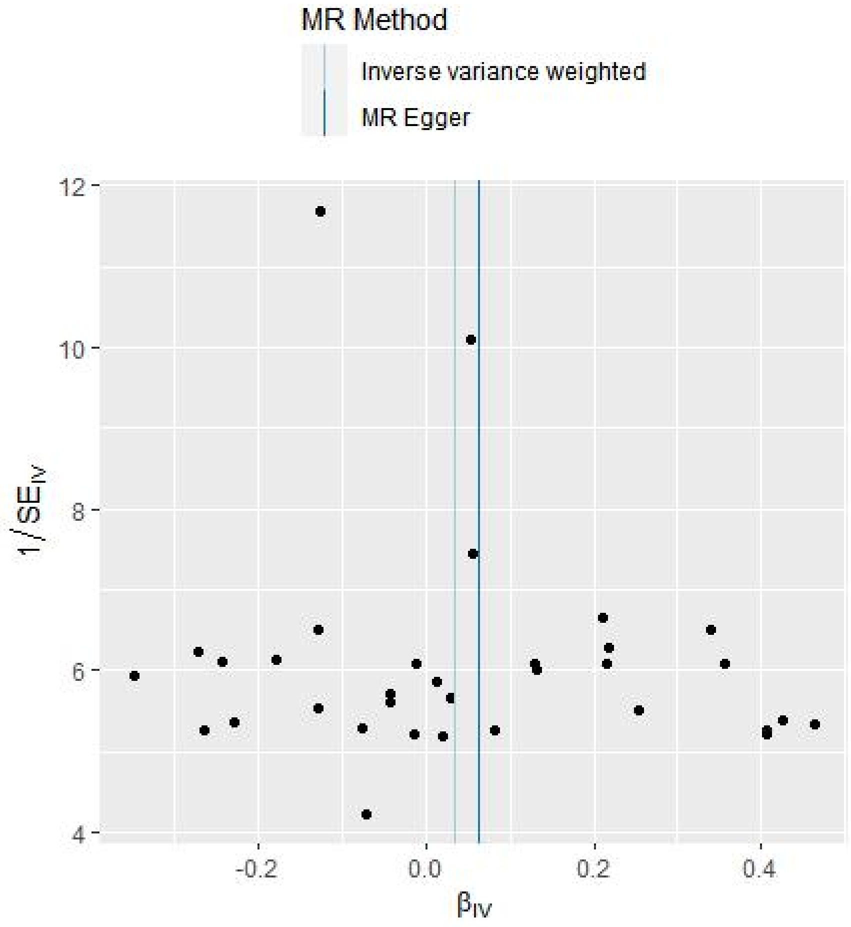

Figure 15: Funnel plots to visualize overall heterogeneity of Mendelian randomization (MR) estimates for the effect of kynuremine on the risk of autism.

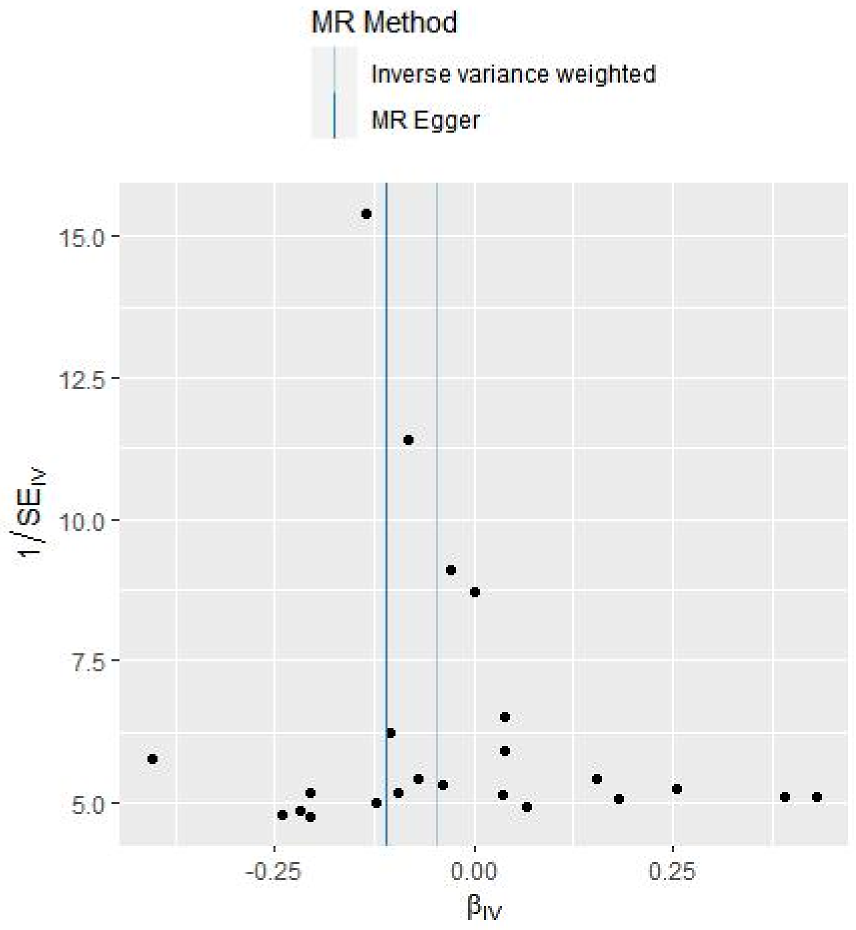

Figure 16: Funnel plots to visualize overall heterogeneity of Mendelian randomization (MR) estimates for the effect of phenylalanine on the risk of autism

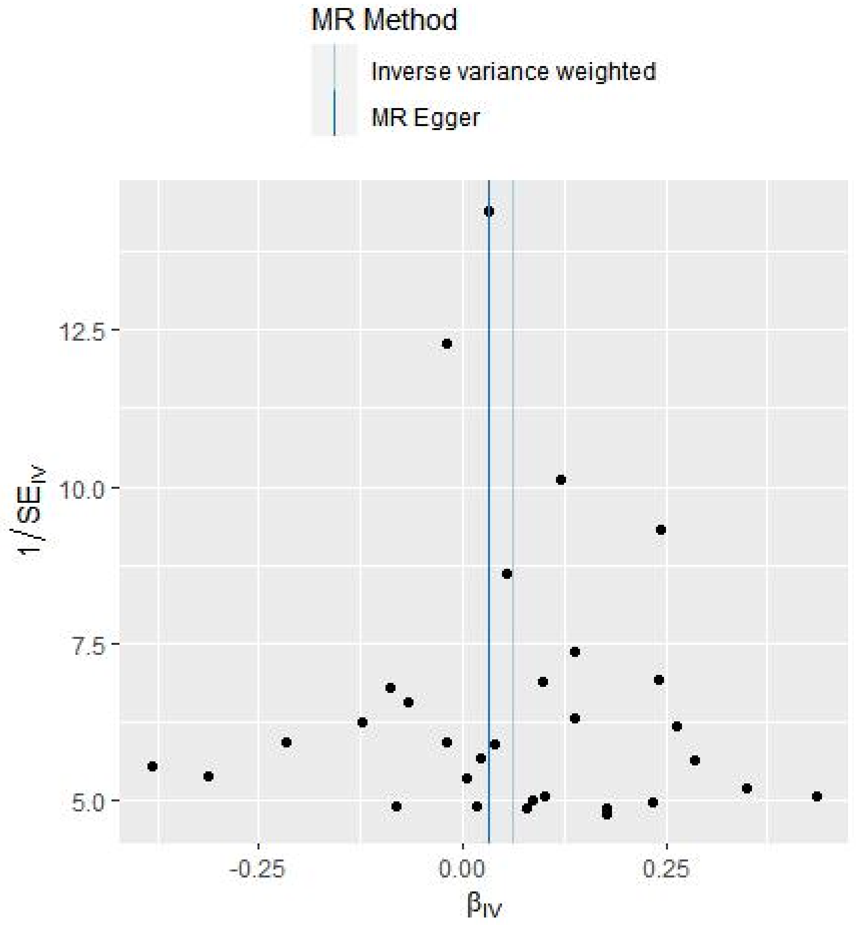

Figure 17: Funnel plots to visualize overall heterogeneity of Mendelian randomization (MR) estimates for the effect of serotonin on the risk of autism

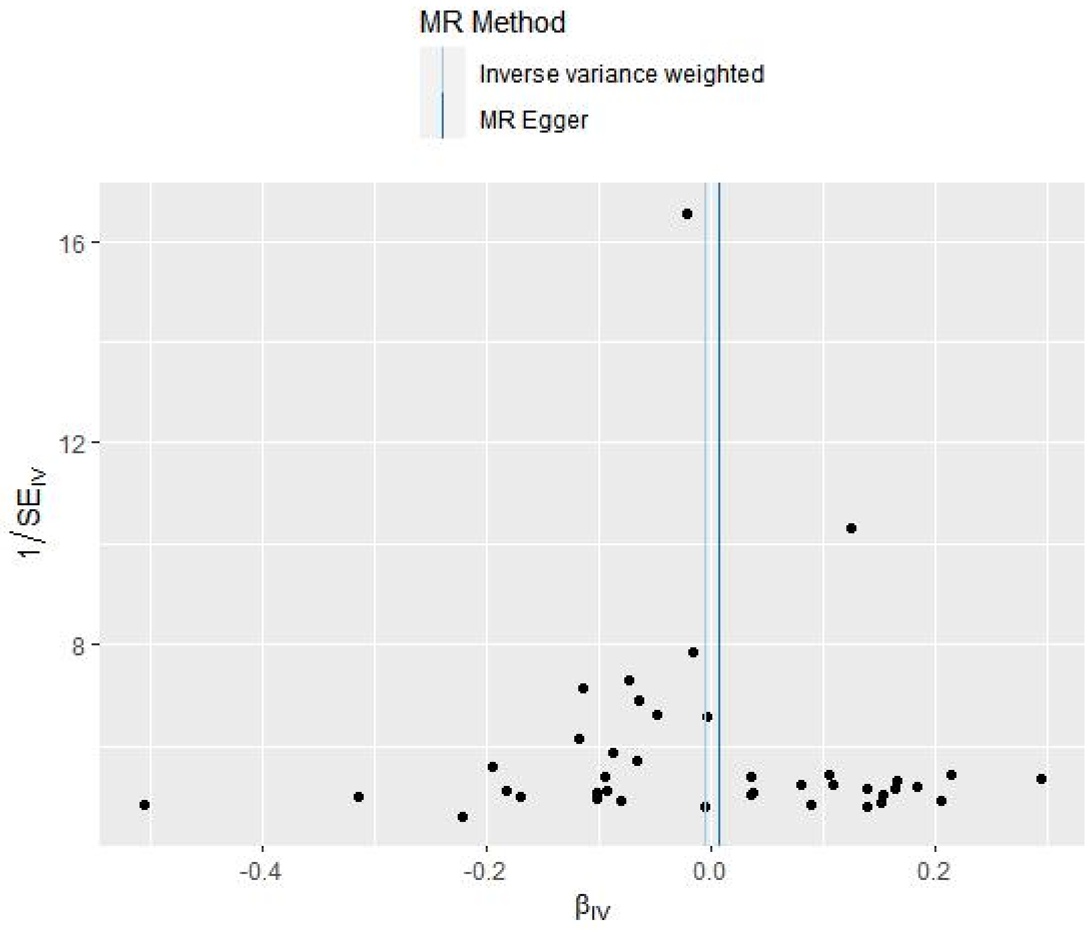

Figure 17: Funnel plots to visualize overall heterogeneity of Mendelian randomization (MR) estimates for the effect of trimethylamine_N_oxide on the risk of autism

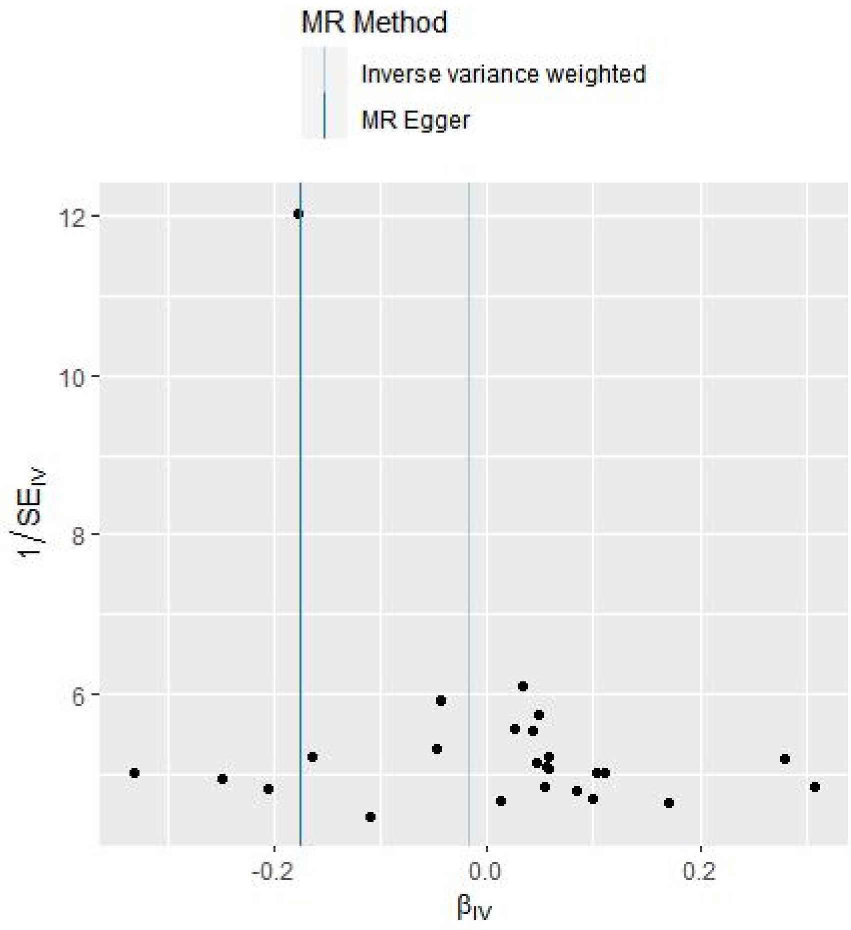

Figure 19: Funnel plots to visualize overall heterogeneity of Mendelian randomization (MR) estimates for the effect of tryptophan on the risk of autism.

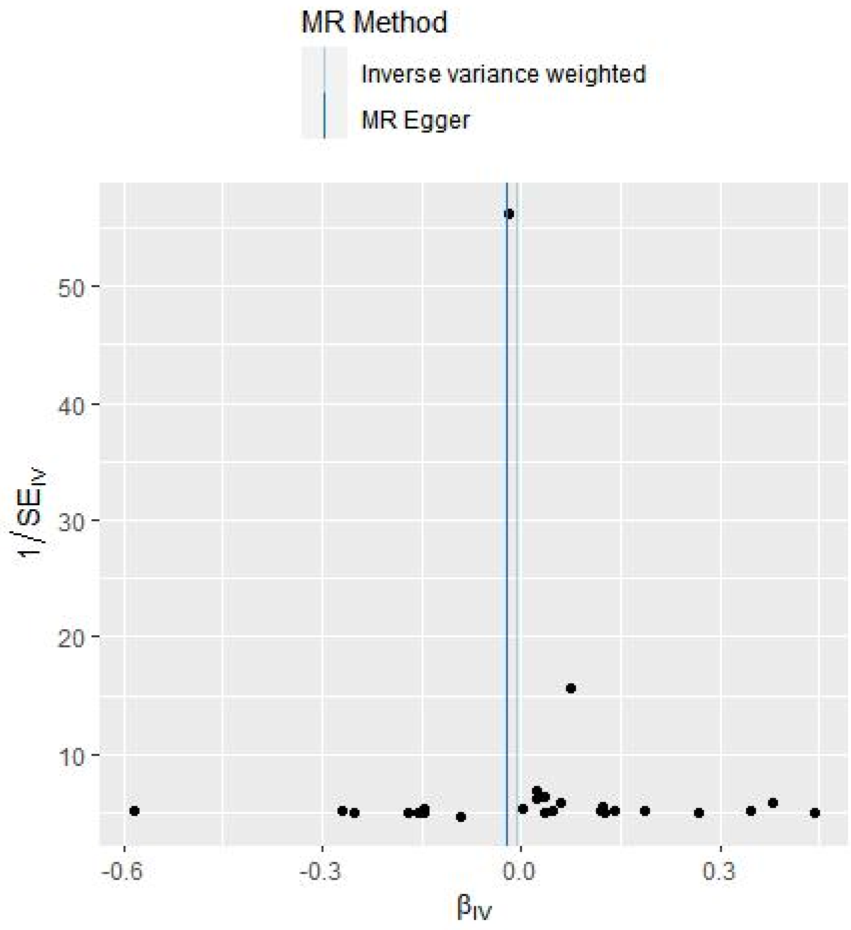

Figure 20: Funnel plots to visualize overall heterogeneity of Mendelian randomization (MR) estimates for the effect of tyrosine on the risk of autism.
